# Data on length-weight relationships and mean condition factor for *Abramis brama* and *Perca fluviatilis*

**DOI:** 10.1016/j.dib.2020.105204

**Published:** 2020-01-25

**Authors:** Victoria V. Yurchenko, Alexey A. Morozov

**Affiliations:** Papanin Institute for Biology of Inland Waters Russian Academy of Sciences, Russia

**Keywords:** LWR, Linear regression, Condition factor, Total weight, Standard length, Fish, Freshwater bream, Perch

## Abstract

Data on total weight and standard length of freshwater bream and perch, two of the most abundant fish species in the Volga River basin, were collected monthly from April 2016 to May 2017. Fish were caught using gill-nets with 50 and 80 mm mesh sizes. Linear regression analysis of log-transformed values was performed. Regression slope and intercept were used to obtain length-weight relationships and mean condition factors. The data can be used for calculation of the relative weights of given species and further efforts to develop a mathematical model for the Volga River ecosystem.

**Table:** Specifications Table

Subject	Animal Science and Zoology
Specific subject area	Ichthyology, fisheries
Type of data	Table, graph
How data were acquired	A measuring board and an OHAUS Navigator NVL2101 digital balance (OHAUS Corporation, USA) were used to acquire raw data. Analyses were performed using Microsoft Office Excel software.
Data format	Raw, analysed, filtered
Parameters for data collection	*Abramis brama* and *Perca fluviatilis*, two of the most abundant in the area [1] and highly commercial fish species [2], were chosen for data collection. Data were collected monthly from April 2016 to May 2017 at one sampling site. Fish were caught by gill-nets with 50 and 80 mm mesh sizes.
Description of data collection	Total weight and standard length of fish were recorded. Length-weight relationships were calculated by the linear regression of log-transformed total weight on standard length. The condition factor was calculated using parameters of length-weight relationships.
Data source location	Confluence of Ild and Sutka rivers (58°07ʹ N 38°27ʹ E), Volga River basin, Yaroslavl Region, Russia
Data accessibility	With the article

**Table:** 

**Value of the Data** • The data contribute to the knowledge of given fish species biology and provide new cases for meta-analysis of length-weight data. • The data can be used for calculation of the relative weight *W*_rm_ [3] for comparing the condition of individuals across populations of given species. • The data are beneficial for further efforts to model the Volga River ecosystem.

## 1 Data

**Fig. 1:**
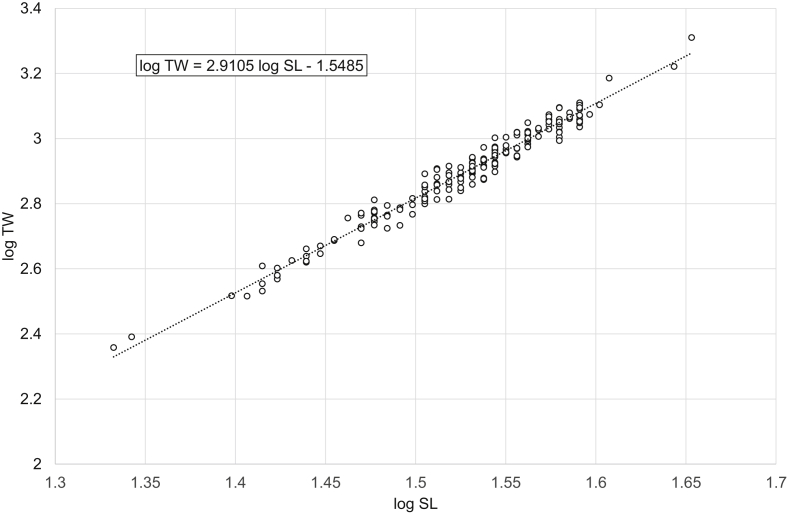
Linear regression analysis of log-transformed SL and TW of freshwater bream *Abramis brama* (pooled sample).

**Fig. 2:**
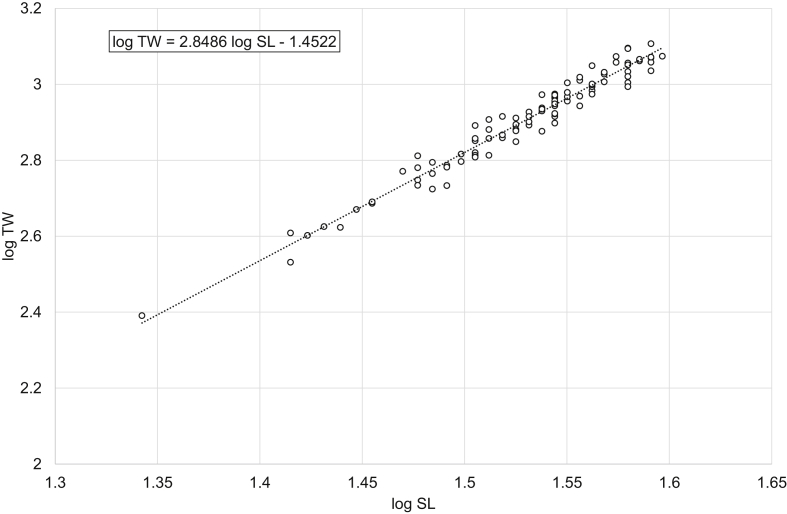
Linear regression analysis of log-transformed SL and TW of freshwater bream *Abramis brama* (males).

**Fig. 3:**
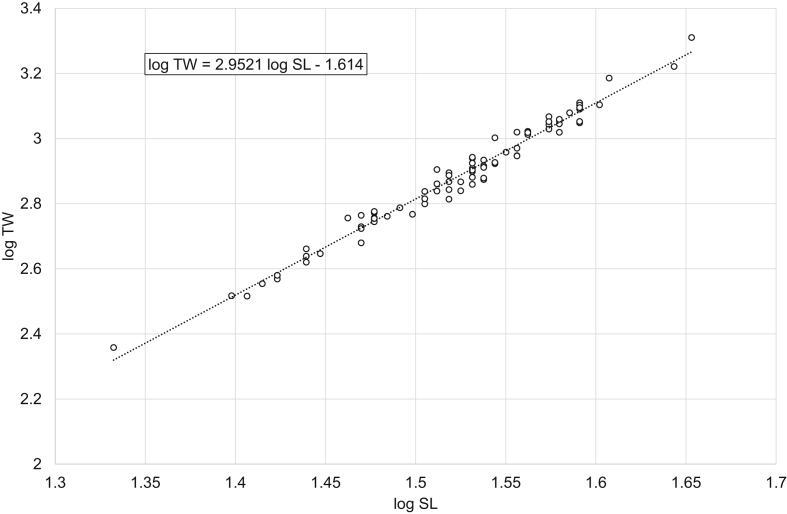
Linear regression analysis of log-transformed SL and TW of freshwater bream *Abramis brama* (females).

**Fig. 4:**
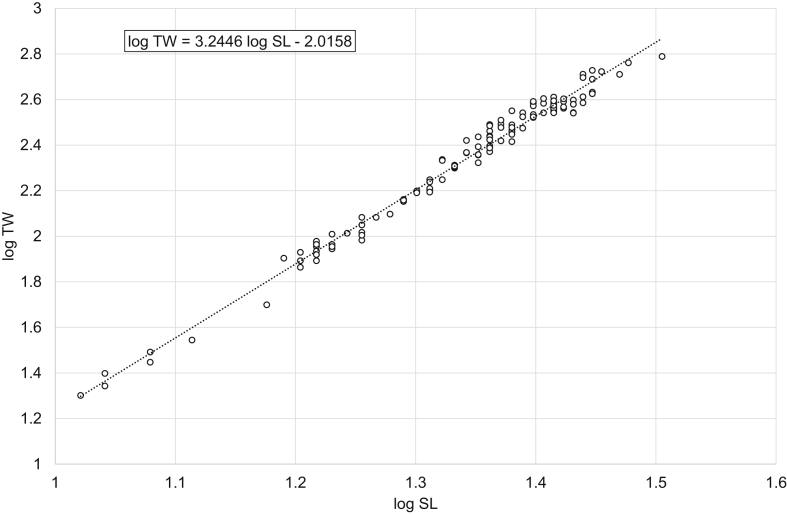
Linear regression analysis of log-transformed SL and TW of perch *Perca fluviatilis* (pooled sample).

**Fig. 5:**
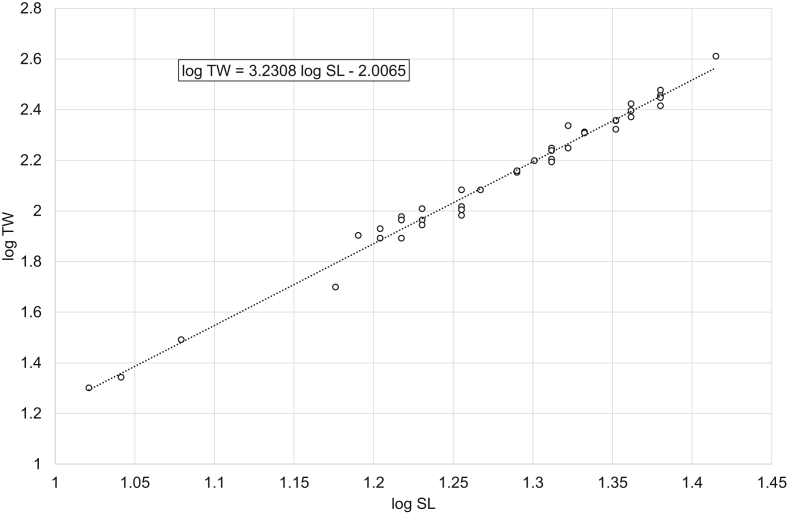
Linear regression analysis of log-transformed SL and TW of perch *Perca fluviatilis* (males).

**Fig. 6:**
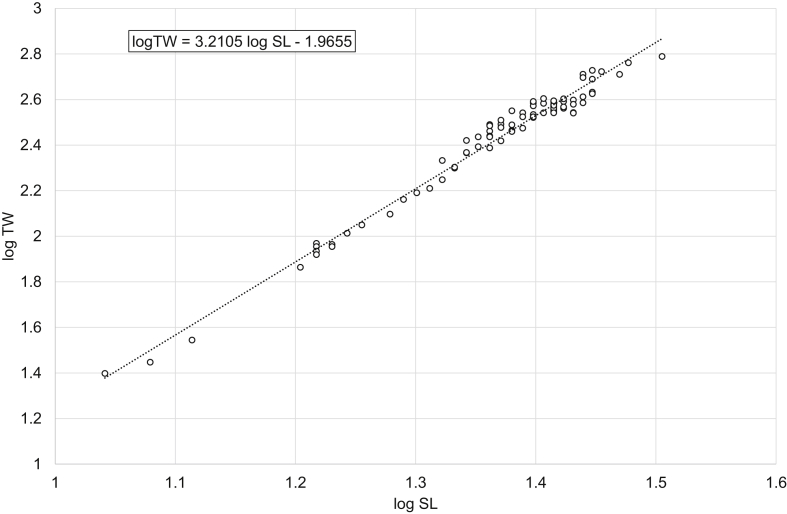
Linear regression analysis of log-transformed SL and TW of perch *Perca fluviatilis* (females).

Fig. 1, Fig. 2, Fig. 3, Fig. 4, Fig. 5, Fig. 6 present linear regression models for the log-transformed raw data on standard length and total weight of freshwater bream *Abramis brama* and perch *Perca fluviatilis*. Descriptive data, linear regression equations, length-weight relationship (LWR) equations, and mean condition factor (CF_mean_) values for the pooled and filtered (males/females) samples are given in Table 1. Raw data related to Fig. 1, Fig. 2, Fig. 3, Fig. 4, Fig. 5, Fig. 6 are reported in spreadsheets and given in the Supplementary Data 1.

**Table 1:** Descriptive data, linear regression analysis, LWR and CF_mean_ of freshwater bream *Abramis brama* and perch *Perca fluviatilis*.

Dataset	*N*	SL, cm		TW, g		Linear regression log TW = *b* log SL + log *a*	*R*^2^	LWR TW = *a* SL^*b*^	CF_mean_ = 100 *a* SL^*b*−3^	
		Min	Max	Min	Max				Min	Max
*Abramis brama* pooled	184	21.5	45.0	228	2044	log TW = 2.9105 log SL – 1.5485	0.9698	TW = 0.0283 SL^2.9105^	2.0116	2.1491
*Abramis brama* males	100	22.0	39.5	246	1279	log TW = 2.8486 log SL – 1.4522	0.9603	TW = 0.0353 SL^2.8486^	2.0234	2.2108
*Abramis brama* females	84	21.5	45.0	228	2044	log TW = 2.9521 log SL – 1.614	0.9769	TW = 0.0243 SL^2.9521^	2.0268	2.0998
*Perca fluviatilis* pooled	128	10.5	32.0	20	615	log TW = 3.2446 log SL – 2.0158	0.9863	TW = 0.0096 SL^3.2446^	1.7139	2.2509
*Perca fluviatilis* males	41	10.5	26.0	20	408	log TW = 3.2308 log SL – 2.0065	0.9877	TW = 0.0099 SL^3.2308^	1.6951	2.0897
*Perca fluviatilis* females	87	11.0	32.0	25	615	log TW = 3.2105 log SL – 1.9655	0.9817	TW = 0.0108 SL^3.2105^	1.7935	2.2456

Note: *N* – number of specimens used for the linear regression analysis; SL – standard length; TW – total weight; *R*^2^ – coefficient of determination of the linear regression; LWR – length-weight relationship; CF_mean_ – mean condition factor for a given length derived from the respective LWR.

## 2 Experimental design, materials, and methods

Sampling was performed each month from April 2016 to May 2017 at the confluence of Ild and Sutka rivers, Volga River basin, using gill-nets with 50 and 80 mm mesh sizes. Fish were treated according to the procedure for use of fishes in the research of the Laboratory of Physiology and Toxicology of Aquatic Animals, Papanin Institute for Biology of Inland Waters Russian Academy of Sciences, consistent with the EU Directive 2010/63/EU for animal experiments [4]. Each individual, one after the other, was immobilized by a stunning blow to the head, quickly measured in standard length (SL) and total weight (TW) and subjected to cervical transection by an experienced person. SL was measured to the nearest 0.5 cm using a measuring board. TW was measured with an accuracy of 1 g using an OHAUS Navigator NVL2101 digital balance (OHAUS Corporation, USA). Each specimen was then dissected and the sex was determined visually.

In total, raw data on 187 specimens of *A. brama* and 129 specimens of *P. fluviatilis* were collected. LWRs were calculated by the linear regression of log-transformed total weight on standard length [1]. Microsoft Office Excel was used for processing the data. Log_10_ values of SL and TW were used to draw a scatter plot for each species. A trend line was added to each scatter plot and visual inspection of outliers was performed. After the outliers were removed manually (three dots in the *A. brama* scatter plot and one in the *P. fluviatilis* scatter plot), final linear-regression equations (log TW = *b* log SL + log *a*) were obtained. Log *a* values were back-transformed to obtain LWRs (TW = *a* SL^*b*^). CF_mean_ values were calculated according to the formula: CF_mean_ = 100 *a* SL^*b*−3^ [3]. In addition, raw data were filtered by the sex criterion, and the appropriate calculations were made for males and females.
